# Supplementary material for: Examining non-reinforced lever pressing as intrinsically-motivated behavior
Source: Anim Cogn. 2026 Jun 3;29(1):54. doi: 10.1007/s10071-026-02066-0 (PMC13453994; doi:10.1007/s10071-026-02066-0)
Supplement: Supplementary file 1 [file 10071_2026_2066_MOESM1_ESM.docx]

**Supplementary Material 1**

**Examining Non-reinforced Lever Pressing as Intrinsically Motivated Behavior**

Sara R. Bond, Jordan Nerz, Katie Cagney, & Kenneth J. Leising

Department of Psychology, Texas Christian University, 2800 S. University Dr., Box 298920, Fort Worth, TX 76129. E-mail: [k.j.leising@tcu.edu](mailto:k.j.leising@tcu.edu)

**Lever press difference score during the manipulation phase of Experiment 1 by Sex**

Lever press rate difference score (Lever Press Rate (per min) – Initial Baseline Rate) is displayed for lever pressing across sessions of the manipulation sessions (5 and 6) for Experiment 1 by group and sex. Positive difference scores indicate elevation of lever press rate relative to the initial baseline period, whereas negative difference scores would indicate a reduction in rate. Session 4 was the magazine training phase, conducted without the lever present in the chamber. Error bars represent standard error of the mean (SEM).

**Supplementary Material 2**

**Examining Non-reinforced Lever Pressing as Intrinsically Motivated Behavior**

Sara R. Bond, Jordan Nerz, Katie Cagney, & Kenneth J. Leising

Department of Psychology, Texas Christian University, 2800 S. University Dr., Box 298920, Fort Worth, TX 76129. E-mail: [k.j.leising@tcu.edu](mailto:k.j.leising@tcu.edu)

**Latency of first response in Experiment 1**

The median latency of the first response during each period of the experiment by group. Sessions 1–3 represent the initial baseline period, while Sessions 5–6 (“Manip.”) represent the manipulation period, and Sessions 7–18 represent the final baseline period. Session 4 was the magazine training phase, conducted without the lever present in the chamber.

**Supplementary Material 3**

**Examining Non-reinforced Lever Pressing as Intrinsically Motivated Behavior**

Sara R. Bond, Jordan Nerz, Katie Cagney, & Kenneth J. Leising

Department of Psychology, Texas Christian University, 2800 S. University Dr., Box 298920, Fort Worth, TX 76129. E-mail: [k.j.leising@tcu.edu](mailto:k.j.leising@tcu.edu)

**Latency of first response in Experiment 2**

The median latency of the first response during each period of the experiment by group. Sessions 1–3 represent the initial baseline period, while Sessions 5–6 (“Manip.”) represent the manipulation period, and Sessions 7–18 represent the final baseline period. Session 4 was the magazine training phase, conducted without the lever present in the chamber.
